# Evaluation of real-time tracheal ultrasound versus colorimetric capnography as a point of care tool for confirmation of endotracheal intubation: a randomized controlled study

**DOI:** 10.1186/s42077-020-00117-3

**Published:** 2020-11-23

**Authors:** Tamer Nabil Abdelrahman, Gamal A. Abdelhameed, Simon H. Armanious

**Affiliations:** Anesthesia, Intensive care and pain management, Faculty of Medicine, Ain Shams University, Cairo, Egypt

**Keywords:** Tracheal ultrasound, Colorimetric capnography, Endotracheal tube

## Abstract

**Background:**

Endotracheal intubation is essential for optimal airway control during general anesthesia or resuscitation of critically ill patients. Misplacement of the endotracheal tube (ETT) can lead to devastating, preventable morbidity and mortality. Colorimetric carbon dioxide detectors are considered a simplistic mainstream capnography that contains a pH-sensitive chemical marker that is prone to color change with ventilation. Real-time tracheal ultrasound allows for the dynamic observation of tube passage through the trachea or the esophagus, providing immediate confirmation of placement prior to any ventilation attempts with reported sensitivity/specificity of 100% for adult patients in the operating room.

We aimed to compare two different techniques (tracheal real-time ultrasound vs colorimetric capnography) as point of care tools for confirmation of correct endotracheal tube position.

**Results:**

This study carried out on eligible seventy patients undergoing general anesthesia. Patients were divided randomly and equally into two groups (35 patients each). Group A in which patients’ ETT position was confirmed by real-time tracheal ultrasound and group B in which patients’ ETT position was confirmed by colorimetric capnography. Comparing both groups according to their diagnostic performance for detecting the correct position of the ETT inserted showed the diagnostic sensitivity, specificity, and accuracy of real-time tracheal ultrasound vs colorimetric capnography (93.8%, 66.7%, and 91.4% VS 97%, 50%, and 94.3% respectively). Although there were higher sensitivity and accuracy of colorimetric capnography than real-time tracheal ultrasound, the *p* value between the two groups was 0.462.

**Conclusion:**

Both tools are fast, effective, reliable, and accurate with many advantages and few disadvantages including the need for training on ultrasound practice and air-filled stomach in colorimetric capnography. However, these disadvantages can be easily overruled and the benefits from both tools overweigh their disadvantages.

## Background

Endotracheal intubation is essential for optimal airway control during general anesthesia or resuscitation of critically ill patients. Unfortunately, clinical signs are not fully sensitive to diagnose esophageal intubation and even risky to rely on alone; thus, other techniques to confirm the proper site of the endotracheal tube (ETT) are devastatingly necessary (Clyburn and Rosen 1994). Misplacement of the ETT can lead to devastating, preventable morbidity, and mortality. The end-tidal carbon dioxide (ETCO_2_) detector used in the operating room is considered a standard of care by anesthesiologists (Ginsburg 1993).

Capnography refers to the measurement of carbon dioxide in the respiratory gases of mechanically ventilated patients. The 2010 American Heart Association (AHA) Guidelines for cardiopulmonary resuscitation (CPR) guidelines and Emergency Cardiovascular Care (ECC) recommended quantitative waveform capnography for verifying the correct placement of an ETT as the gold standard (Neumar et al. 2010). There are two types of sampling techniques in capnographic devices: mainstream or sidestream. The mainstream analyzer deploys a sampling window in the ventilation circuits, whereas a sidestream analyzer samples gas from the ventilator circuit, and the analysis occurs away from the ventilator circuit. Analyzers utilize infrared, mass or Raman spectra, or photoacoustic spectra technology (Block Jr and McDonald 1992; O’Flaherty 1994). Colorimetric carbon dioxide detectors are considered a simplistic mainstream capnography that contains a pH-sensitive chemical marker that is prone to color changes with each inspiration and expiration representing changes in the concentration of carbon dioxide. These devices start at baseline color when minimal carbon dioxide is present and undergo gradual color change with increasing carbon dioxide concentration (O’Flaherty 1994).

Capnography is less reliable in the setting of cardiac arrest patients and can be affected by low cardiac output, low pulmonary flow, airway obstruction, or epinephrine use in addition to its unavailability outside the operating theater. (Bhende and Thompson 1995; Zechner and Breitkreutz 2011) In one prospective study, the sensitivity of this device in detecting proper endotracheal placement in cardiac arrest patients was only 85% (Takeda et al. 2003).

Point-of-care ultrasound is defined as ultrasonography brought to the patient and both performed and interpreted “real-time” by the provider. It is quick and inexpensive, and with the recent development of handheld ultrasound devices, it is already readily available in the clinical areas where endotracheal intubation occurs. (Moore and Copel 2011) Real-time tracheal ultrasound allows for the dynamic observation of tube passage through the trachea or the esophagus, providing immediate confirmation of placement prior to any ventilation attempts with reported sensitivity/specificity of 100% for adult patients in the operating room (Muslu et al. 2011a) and 100%/86%, respectively, in patients undergoing cardiopulmonary resuscitation (Chou et al. 2013). For sonographic assessment of the ETT position, previous investigators have used several different methods, including tracheal ultrasonography (direct), and detecting sliding of the pleura or diaphragm movement (indirect) (Chou et al. 2011; Sim et al. 2012).

### Aim of the work

We aimed to compare two different techniques (tracheal real-time ultrasound vs colorimetric capnography) as point of care tools for confirmation of correct endotracheal tube position.

## Methods

This is a prospective, randomized, controlled study carried out on eligible patients (*n* = 70) undergoing general anesthesia. Randomization was performed with the help of a computer-generated list of numbers. This study was approved by the local institutional ethical committee board (FAMSU R 4/2020). Written informed consent was obtained from all patients before the study. This research was registered in the Pan African Clinical Trials Registry (https://pactr.samrc.ac.za/) in the 2nd of March 2020 with the following ID (PACTR 202003901).

Patients were divided randomly and equally into two groups (35 patients each). Group A (U.S group) in which patients’ ETT position was confirmed by real-time tracheal ultrasound and group B (colorimetric capnography group) in which patients’ ETT position was confirmed by colorimetric capnography. For patients with esophageal intubation, the first intubation attempt was only included in the study while the next intubation trials were not included.

### Study population

Participants of the study should be ≥ 18 years of age, fasting for at least 6 h before anesthesia, and underwent general anesthesia with ETT insertion. While we excluded patients who were not fasting, pregnant females, history of recent cervical trauma, any midline neck swelling, and patients risky for difficult intubation with (Mallampati score more than or equal to 3, a thyromental distance less than 6 cm, neck circumference more than 40 cm) or history of a difficult airway.

### Study methodology

All candidates were subjected to a thorough medical history, physical examination with airway assessment, laboratory investigations (fasting blood sugar, kidney, liver function tests, serum electrolytes, coagulation profile, and electrocardiogram) preoperatively, and informed consent was obtained. All participants were admitted to O.R. where general anesthesia started with preoxygenation and induction of sleep with intravenous injection of propofol 2 mg/kg, atracurium 0.5 mg/kg for muscle relaxation, fentanyl 2 μg/kg. Then, a suitable ETT for gender was inserted using a direct laryngoscope by anesthetist and confirmation of the ETT position by the two studied techniques and the position of ETT was further assured in all patients by using infra-red capnography and chest auscultation to support the data delivered in either group.

### Group A (U.S group)

**Fig. 1:**
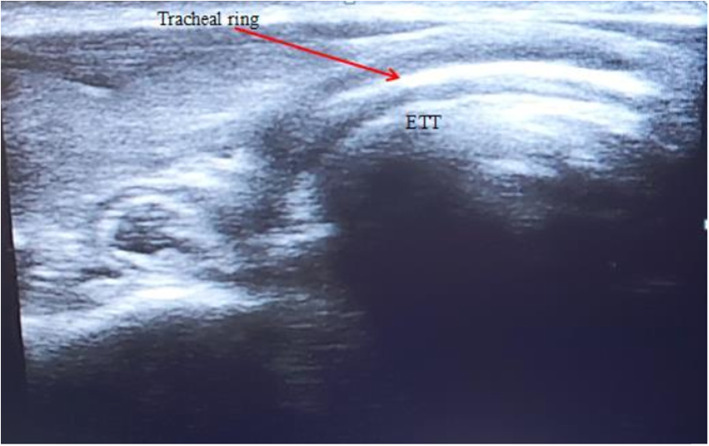
Midline ultrasonographic view of the trachea with the endotracheal tube (ETT) in the right position

The ETT position was confirmed by “real-time ultrasound” using a high-frequency linear probe” (sonosite M-Turbo®) over the midline of the neck in the suprasternal notch:

- The sonographer can watch as the tube passes into the trachea. Motion will be visible just posterior to the tracheal cartilage during intubation. This is seen as an area of increased echogenicity.
- Additionally, the sonographer should watch for the appearance of a second air-mucosal interface adjacent to the trachea, which would indicate the passage of the ETT into the esophagus. If no second air-mucosal interface is appreciated, the tube has been correctly placed in the trachea (Fig. 1).

### Group B (colorimetric capnography group)

**Fig. 2:**
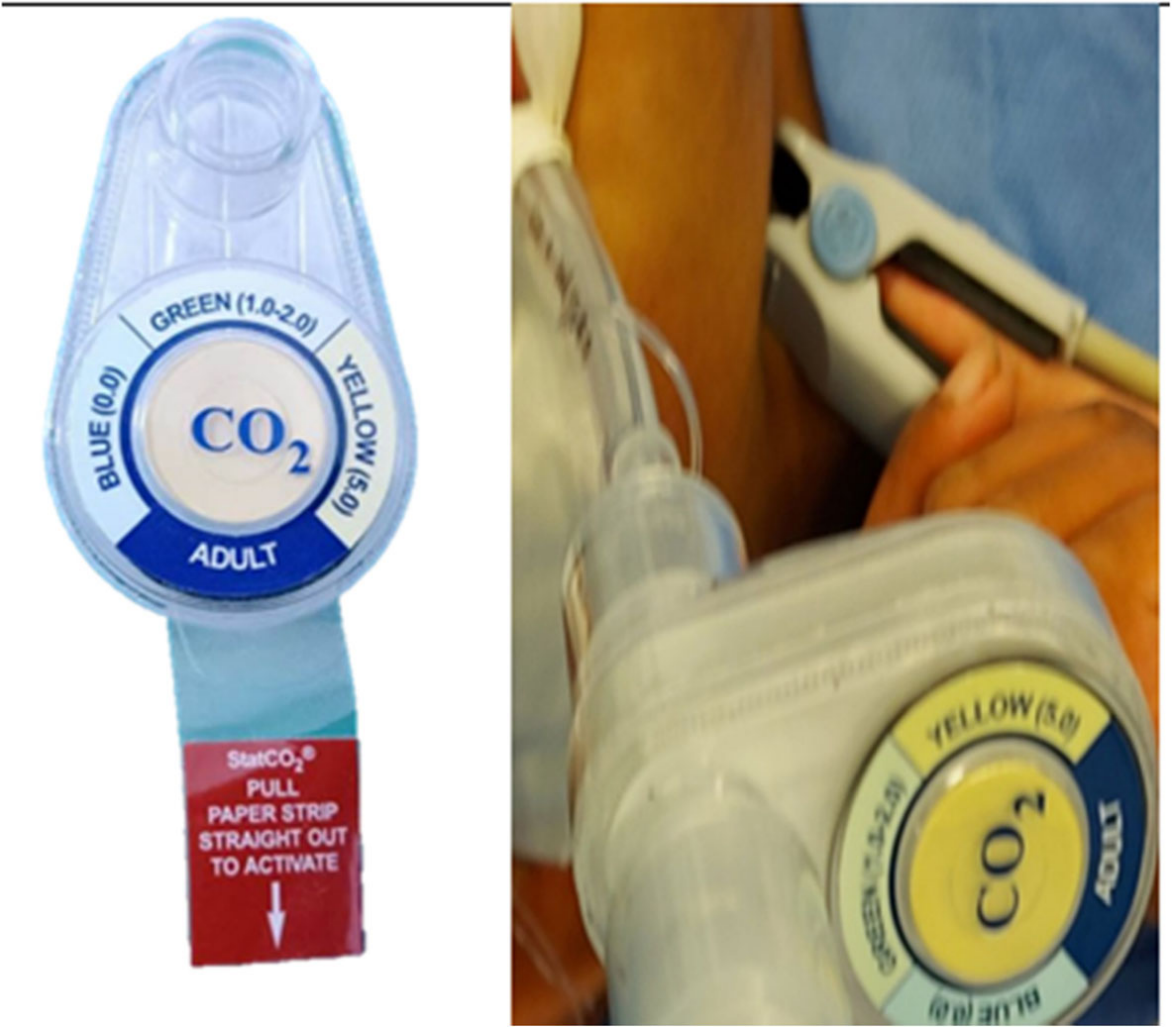
Colorimetric capnography before use in the left image and mounted on the ETT on the right image

The ETT position was confirmed by “colorimetric capnography” (StatCO_2_, Mercury Medical Corp.), where it is first applied to the ETT, and color changes were observed during mechanical ventilation where color ranges are identified as the “A” range (blue): approx. 0.03 to < 0.5% EtCO^2^ (< 4 mm Hg), “B” range (green): approx. 0.5 to < 2.0% EtCO_2_ (4 to < 15 mm Hg), and “C” range (yellow): approx. 2.0 to 5.0% EtCO_2_ (15 to 38 mm Hg) (Fig. 2).

The following will be measured:

The primary outcome was to estimate the sensitivity and specificity of real-time tracheal ultrasound versus colorimetric capnography detector in confirming the position of the ETT using standard waveform capnography and chest auscultation as gold standard references.

The secondary outcome was to measure the time elapsed until both maneuvers confirmed the position of the ETT either tracheal or esophageal.

In case the ETT malposition either oesophageal or bronchial intubation was discovered so immediate repositioning of the ETT was performed by a senior attending anesthesia staff and the second attempt was not included in the study.

## Results

**Fig. 3:**
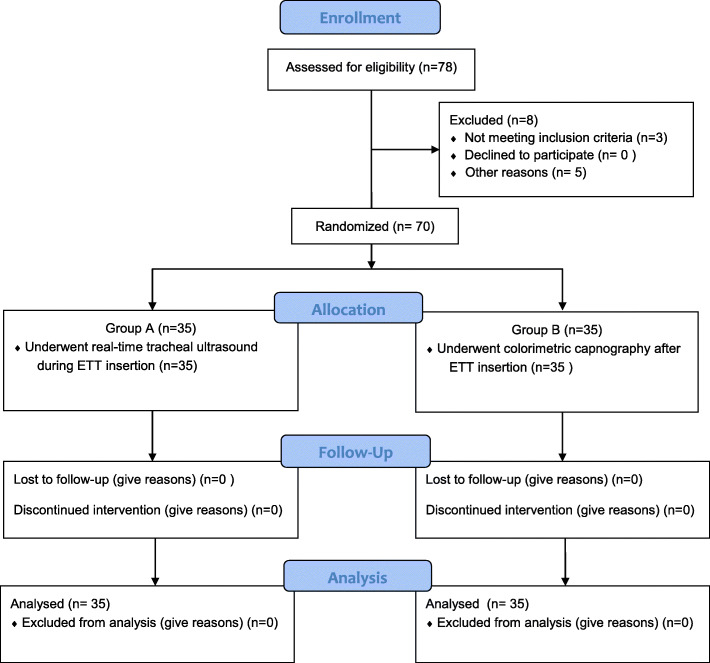
Consort chart of the study

This is a prospective comparative study that included a total of 70 consented adult patients undergoing general anesthesia with endotracheal intubation. Patients were randomly divided equally into two groups the first group A was assigned to “real-time tracheal ultrasound” confirmation of the ETT position (*N* = 35), and the other group B was assigned to “colorimetric capnography” confirmation of the ETT position (*N* = 35). All participants completed the trial (Fig. 3).

Table 1 compares the baseline characteristics of the study participants including the patient age, gender, body mass index (BMI), American Society of Anesthesiology (ASA) physical status, number of unexpected difficult intubation, and average number of attempts for endotracheal intubation. There were statistically insignificant differences between the two groups regarding all previously mentioned aspects (Table 1).

**Table 1:** Comparison between group A (U.S) and group B (colorimetric CO_2_ detector) according to baseline characteristics

Baseline characteristics	Group A (U.S) [***n*** = 35]	Group B (colorimetric CO_**2**_ detector) [***n*** = 35]	Test of significant	***p*** value
**Age (years)**				
Range	21–70	22–70	*t* = 0.411	0.206
Mean ± SD	49.14 ± 13.69	50.86 ± 11.51		
**Sex**				
Male	22 (62.9%)	20 (57.1%)	*X*^2^ = 0.221	0.605
Female	13 (37.1%)	15 (42.9%)		
**BMI [wt/(ht)^2]**				
Range	24.0-31.6	26.5–33.9	*t* = 1.03	0.124
Mean ± SD	27.56 ± 2.31	30.29 ± 2.46		
**ASA**				
I/II	30 (85.7%)	28 (80.0%)	*X*^2^ = 0.114	0.691
III/IV	5 (14.3%)	7 (20.0%)		
**Unexpected difficult intubation**	3 (8.6%)	5 (14.3%)	*X*^2^ = 0.631	0.190
**No. of attempts of intubation**				
Range	1–3	1–3	*t* = 1.15	0.187
Mean ± SD	1.31 ± 0.58	1.43 ± 0.61		

*t* independent sample *t* test, *X*^2^ chi-square test; *p* value > 0.05 NS

**Fig. 4:**
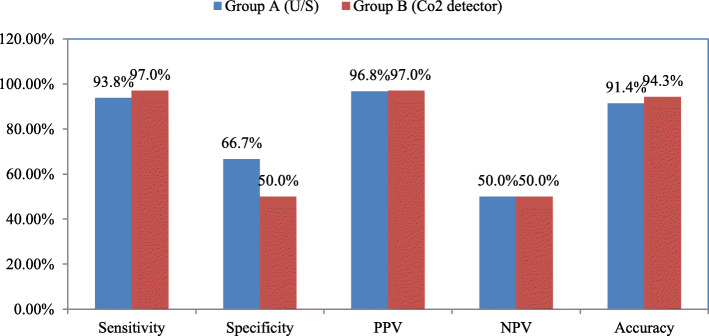
Comparison between group A (U.S group) and group B (colorimetric capnography group) according to their diagnostic performance for detecting the correct position of the ETT position. (PPV positive predictive value, NPV negative predictive value)

Comparing both groups according to their diagnostic performance for detecting the correct position of the ETT inserted showed the diagnostic sensitivity, specificity, and accuracy of real-time tracheal ultrasound vs colorimetric capnography (93.8%, 66.7%, and 91.4% VS 97%, 50%, and 94.3%, respectively). Although there were higher sensitivity and accuracy of colorimetric capnography than real-time tracheal ultrasound, the *p* value between the two groups was 0.462 (Fig. 4).

It was also noted that the time interval needed until the identification of the correct position of the ETT showed a highly significant difference, where colorimetric capnography needed much less time in seconds than real-time tracheal ultrasound (16.35 ± 3.11 Vs 32.7 ± 6.21, respectively, *p* value < 0.001) (Table 2).

**Table 2:** Comparison between group A (U.S) and group B (colorimetric CO_2_ detector) according to time passed till identification of ETT position in seconds

Time passed till identification of ETT	Group A (U/S) [***n*** = 35]	Group B (Colorimetric CO_**2**_ detector) [***n*** = 35]	***t*** test	***p*** value
Range	15–45	10–20	9.381	< 0.001**
Mean ± SD	32.7 ± 6.21	16.35 ± 3.11		

*t* independent sample *t* test, *p* value < 0.001 HS, *Significant

### Statistical analysis

Recorded data were analyzed using the statistical package for social sciences, version 20.0 (SPSS Inc., Chicago, Illinois, USA). Quantitative data are expressed as the mean ± standard deviation (SD). Qualitative data are expressed as frequencies and percentages. Independent samples *t* test of significance was used when comparing between two means, while the chi-square (*X*^2^) test of significance was used in order to compare proportions between qualitative parameters.

Evaluation of the diagnostic performance was performed measuring the following parameters sensitivity, specificity, positive predictive value (PPV), negative predictive value (NPV), and accuracy. The confidence interval was set to 95% and the margin of error accepted was set to 5%. So, the *p* value < 0.05 was considered significant.

### Sample size

The outcome was to identify the sensitivity and specificity of the two techniques in confirming the position of the ETT inserted using two groups. A sample size of at least 26 cases per group was used to achieve 80% power to detect an effect size of at least 0.8 (large effect size) using a two-sided *t* test with a level of significance ≤ 0.05.

## Discussion

Secure management of the airway is crucial for effective successful general anesthesia and/or CPR. Undiagnosed esophageal intubation remains one of the most serious causes of anesthesia-related morbidity and mortality (Salem and Baraka 2007).

The reliability of any tracheal intubation confirmatory technique depends on the frequency of false-positive and negative results. Several instruments and methods for tracheal tube location detection were identified with most depending on indirect measures, such as major physical examination, pulse oximetry, continuous capnography, chest radiography, and fiberoptic bronchoscopy (Bair et al. 2005; Angelotti et al. 2006; Ochroch and Eckmann 2002).

In our study, we examined each method independently and compared them together. Both tests revealed high sensitivity, specificity, and nearly perfect negative likelihood ratios in assessing the ETT position. Our results are consistent with many studies performed on both techniques emphasizing their high sensitivity, as proven by Muslu et al., who proved that tracheal ultrasonography can be as fast as 3 s, highly sensitive, and specific secondary confirmatory methods for endotracheal intubations in patients undergoing elective surgery (Muslu et al. 2011b) and Adi et al., who compared tracheal ultrasonography to waveform capnography in assessing ETT position. They proved that the ulrasonography is a highly accurate method (98.1%). The Kappa value (K) was 0.85 denoting an outstanding agreement between upper airway ultrasonography and waveform capnography. Therefore, the researchers recommended the use of bedside upper airway ultrasonography as a substitute to verify the ETT position because of its simplicity, feasiblity, and quickness (Adi et al. 2013). This finding is consistent with our results in the ultrasound group, where we discovered that the ultrasonography accuracy rate was 94.3% with a sensitivity of 96.9%, specificity of 66.7%, negative predictive value (NPV) of 66.7%, and positive predictive value (PPV) of 96.9%. These results can be explained by building up tracheal ultrasonographic skills and experience with both the machine used and the procedure itself.

Regarding colorimetric capnography, our study showed excellent results as an independent method for confirming ETT position with sensitivity 97%, specificity 50%, and accuracy 94.3%. This goes with previous studies, such as Bhende MS and Thompson AE, who found that colorimetric capnometry was an effective tool in confirmation of the ETT position during pediatric CPR with measured sensitivity was 84.6%, specificity 100%, positive predictive value 100%, and negative predictive value 60% (Bhende et al. 1992). Additionally, Sanders et al. conducted a study at a level I trauma center to assess the efficacy of end-tidal carbon dioxide (CO_2_) detector in four groups of patients requiring emergency intubation. The CO_2_ detector was found to be 100% reliable and useful for confirming ETT placement. So, it can be used effectively during airway management of diverse groups of patients in the emergency settings (Sanders et al. 1994) and Puntervoll et al. compared the colorimetric capnographic indicator to waveform capnography for fast detection of the ETT position and found that both methods verified correct placement of the ETT from the first ventilation. However, waveform capnography was better than colorimetric capnometry in detecting oesophageal intubation rapidly in air-filled stomach situations (Puntervoll et al. 2002).

In our study group B, there was one false-positive result, which was attributed to an air-filled stomach during bag-mask ventilation during the induction of anesthesia.

We concluded that both tools are fast, effective, reliable, and accurate with many advantages and few disadvantages including the need for training on ultrasound practice and air-filled stomach in colorimetric capnography. However, these disadvantages can be easily overruled and the benefits from both tools overweigh their disadvantages.

### Study limitations

Nevertheless, this study had some limitations. There might be subtle biases that can be ignored because of the high rate of successful endotracheal intubation for each patient. Another limitation encountered was an accurate patient selection based on inclusion and exclusion criteria. In addition to a smaller sample size, our findings still need more interpretation for further study. This should be addressed by future prospective studies to verify and clarify the role of both tools in the assessment of the right ETT position.

## Data Availability

All the data of this article are available, from the corresponding author upon request. The email address of the corresponding author is tamernabil610@gmail.com

## Abbreviations

ETCO_2_: End-tidal carbon dioxide
CPR: Cardiopulmonary resuscitation
O.R.: Operating room
ETT: Endotracheal tube
BMI: Body mass index
ASA: American Society of Anesthesiology
PPV: Positive predictive value
NPV: Negative predictive value
U.S: Ultra-sonography
CO_2_: Carbon dioxide

## References

[CR1] Adi O, Chuan TW, Rishya M (2013). Feasibility study on bedside upper airway ultrasonography compared to waveform capnography for verifying endotracheal tube location after intubation. Crit Ultrasound J.

[CR2] Angelotti T, Weiss EL, Lemmens HJM, Brock-Utne J (2006). Verification of endotracheal tube placement by prehospital providers: is a portable fiberoptic bronchoscope of value?. Air Med J.

[CR3] Bair AE, Smith D, Lichty L (2005). Intubation confirmation techniques associated with unrecognized non-tracheal intubations by pre-hospital providers. J Emerg Med.

[CR4] Bhende MS, Thompson AE (1995). Evaluation of an end-tidal carbon dioxide detector during pediatric cardiopulmonary resuscitation. Pediatrics.

[CR5] Bhende MS, Thompson AE, Cook DR, Saville AL (1992). Validity of a disposable End-Tidal CO_2_ detector in verifying endotracheal tube placement in infants and children. Ann Emerg Med.

[CR6] Block FE, McDonald JS (1992). Sidestream versus mainstream carbon dioxide analyzers. J Clin Monit.

[CR7] Chou HC, Chong KM, Sim SS (2013). Real-time tracheal ultrasonography for confirmation of endotracheal tube placement during cardiopulmonary resuscitation. Resuscitation.

[CR8] Chou HC, Tseng WP, Wang CH (2011). Tracheal rapid ultrasound exam (T.R.U.E.) for confirming endotracheal tube placement during emergency intubation. Resuscitation.

[CR9] Clyburn P, Rosen M (1994). Accidental oesophageal intubation. Br J Anaesth.

[CR10] Ginsburg WH (1993). When does a guideline become a standard? The New American Society of Anaesthesiologist’s guidelines give us a clue. Acad Emerg Med.

[CR11] Moore CL, Copel JA (2011) Point-of-care ultrasonography. N Engl J Med:749–757. 10.1056/nejmra0909487 10.1056/NEJMra0909487 21345104

[CR12] Muslu B, Sert H, Kaya A (2011). Use of sonography for rapid identification of esophageal and tracheal intubations in adult patients. J Ultrasound Med.

[CR13] Neumar RW, Otto CW, Link MS (2010). Adult advanced cardiovascular life support: 2010 American Heart Association Guidelines for Cardiopulmonary Resuscitation and Emergency Cardiovascular Care. Part 8. 1: adjuncts for airway control and ventilation. Circulation.

[CR14] O’Flaherty D (1994). Capnometry.

[CR15] Ochroch EA, Eckmann DM (2002). Clinical application of acoustic reflectometry in predicting the difficult airway. Anesth Analg.

[CR16] Puntervoll SA, Søreide E, Jacewicz W, Bjelland E (2002). Rapid detection of oesophageal intubation: take care when using colorimetric capnometry. Acta Anaesthesiol Scand.

[CR17] Salem MR, Baraka A, Hagberg CA (2007). Confirmation of tracheal intubation. Benumof’s Airway Management: Principles and Practice.

[CR18] Sanders KC, Clum WB, Nguyen SS (1994). End-tidal carbon, dioxide detection in emergency intubation in four groups of patients. J Emerg. Med.

[CR19] Sim SS, Wan-Ching L, Chou HC (2012). Ultrasonographic lung sliding sign in confirming proper endotracheal intubation during emergency intubation. Resuscitation.

[CR20] Takeda T, Tanigawa K, Tanaka H (2003). The assessment of three methods to verify tracheal tube placement in the emergency setting. Resuscitation.

[CR21] Zechner PM, Breitkreutz R (2011). Ultrasound instead of capnometry for confirming tracheal tube placement in an emergency?. Resuscitation..

